# Prevalence of Metabolic Syndrome in Relation to Cardiovascular Biomarkers and Dietary Factors among Adolescents with Type 1 Diabetes Mellitus

**DOI:** 10.3390/nu14122435

**Published:** 2022-06-12

**Authors:** Monika Grabia, Renata Markiewicz-Żukowska, Katarzyna Socha, Agnieszka Polkowska, Aneta Zasim, Karolina Boruch, Artur Bossowski

**Affiliations:** 1 Department of Bromatology, Faculty of Pharmacy with the Division of Laboratory Medicine, Medical University of Białystok, Mickiewicza 2D Street, 15-222 Białystok, Poland; monika.grabia@umb.edu.pl (M.G.); katarzyna.socha@umb.edu.pl (K.S.); 2 Clinic of Paediatrics, Endocrinology, Diabetology with Subdivision of Cardiology, Children’s University Clinical Hospital in Białystok, 15-274 Białystok, Poland; inezpol@gmail.com (A.P.); aneta.zasim@wp.pl (A.Z.); abossowski@hotmail.com (A.B.); 3 Clinic of Paediatrics, Rheumatology, Immunology and Bone Metabolic Diseases, Children’s University Clinical Hospital in Białystok, 15-274 Białystok, Poland; karolina.boruch@o2.pl

**Keywords:** diabetes type 1, adolescents, metabolic syndrome, obesity, insulin therapy, nutritional status, nutrients intake, antioxidant status, body composition, continuous glucose monitoring

## Abstract

The occurrence of metabolic syndrome (MetS) significantly affects the course of diabetes mellitus (DM), resulting in deterioration of insulin sensitivity and metabolic control, as well as many cardiometabolic complications. The aim of the study was to investigate the relationships between cardiovascular biomarkers, nutritional status, dietary factors and the occurrence of MetS among 120 participants from northeast Poland (adolescents with type 1 DM and healthy peers). MetS was assessed using several criteria: nutritional status by anthropometric measurements, body composition analysis by bioelectrical impedance, and diet using a food diary and questionnaire. MetS was diagnosed in every third diabetic. Compared to healthy peers, MetS patients had higher total body fat (26% vs. 14%, *p* < 0.001) and visceral fat (77 cm^2^ vs. 35 cm^2^, *p* < 0.001), and lower total antioxidant status (1.249 mmol/L vs. 1.579 mmol/L, *p* < 0.001). Additionally, their diet was rich in saturated fatty acids, but low in dietary fiber as well as mono- and polyunsaturated fatty acids. The group of diabetics reported many inappropriate eating behaviors. The combination of those with the presence of an excessive content of visceral fat tissue and abnormal values of MetS components may negatively affect metabolic control, thus accelerating the development of cardiometabolic complications.

## 1. Introduction

Diabetes mellitus (DM) has become one of the most common chronic diseases of the 21st century [1]. Type 1 diabetes mellitus (T1DM) is an autoimmune disease in which the body attacks the beta cells of the pancreas, which produce insulin. As a result, insufficient amounts of insulin are secreted. The disease is most common in children and adolescents [2]. In 2021, the worldwide incidence of DM reached the level of 1.2 million children and adolescents up to the age of 19, exceeding 180,000 new diagnoses annually [1]. Patients need daily insulin injections to keep their blood glucose levels within the proper range. Intensive insulin therapy can be performed by means of continuous subcutaneous insulin infusion (CSII) with an insulin pump or multiple daily insulin-pen injections (MDI) [2]. Flash (FGM) or continuous (CGM) glucose-monitoring systems are becoming increasingly popular methods of monitoring blood glucose levels. They are designed to facilitate everyday life and reduce the number of punctures of fingertips, as well as the number of hypo- and hyperglycaemic incidents [3].

Increasingly, overweight and obesity are being diagnosed as resulting from poor eating habits and low physical activity [4,5]. In combination, they can significantly worsen metabolic management, contributing to the development of metabolic syndrome (MetS) defined as a set of multiple biochemical, metabolic, and physiological factors that increase the risk of many cardiometabolic complications [6].

The most common diagnostic criteria used by researchers are created by the International Diabetes Federation (IDF) [7], the National Cholesterol Education Program Adult Treatment Panel III (ATP) [8], and the World Health Organization (WHO) [9]. Depending on the selection, estimates are quite divergent (between 3% and 30% of the pediatric population has T1DM). Although adult T1DM is widely covered by the literature, there is little research on this disease among children and adolescents, especially in conjunction with the analysis of their nutritional status and diet. Guidelines use different values of the criteria with different degrees of severity for the individual components of MetS. In addition, some of the criteria do not take age into account and, therefore, often differ from the national standards for the physical development of the pediatric population. Moreover, there is a dearth of studies that include comparative analyses of control groups [10].

The aim of the study was to investigate the relationships between the occurrence of MetS and cardiovascular biomarkers, nutritional status, and dietary factors among adolescents with T1DM, in comparison to healthy peers.

## 2. Materials and Methods

### 2.1. Study Group

**Figure 1:**
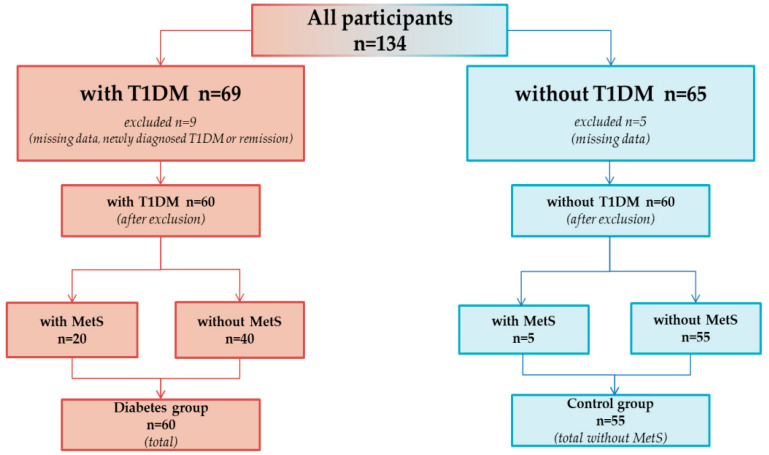
Flowchart of the inclusion process in the study.

The case-control study was conducted among 120 Polish patients with T1DM and healthy adolescents aged 10–17 years between March 2020 and July 2021. The T1DM group (*n* = 60) contained adolescents with T1DM from the Clinic of Pediatrics, Endocrinology, Diabetology with Subdivision of Cardiology, in the Children’s University Clinical Hospital in Białystok, while the group without T1DM (*n* = 60) consisted of adolescents who reported directly to the Department of Bromatology, in the Medical University of Białystok. The recruitment process for the control group involved an interview to verify that they had no symptoms indicating the possibility of diabetes or other chronic diseases. In addition, the questionnaire included a screening question about the presence of various chronic diseases. The inclusion process is illustrated in Figure 1. The main study-inclusion criteria were: age between 10 and 17 years as well as T1DM and diabetes lasting for more than two years, without remission. The diagnosis of T1DM was confirmed according to the guidelines of the American Diabetes Association, with consideration of the frequent presence of autoantibodies (glutamic acid decarboxylase, islet cell antibodies, and insulin autoantibodies) in blood samples [11]. Written consent of the participants’ parents and the bioethics committee (No. R-I-002/587/2019) were obtained to perform the study.

### 2.2. Blood Samples Analysis of Cardiovascular Biomarkers

Blood samples were drawn from participants using vacutainer-system tubes containing clot activator + gel or anticoagulant K2EDTA (Becton Dickinson, France). The material was appropriately prepared and tested shortly after collection (total cholesterol (TC), low-density lipoprotein cholesterol (LDL-ch), high-density lipoprotein cholesterol (HDL-ch), triglycerides (TG), fasting glucose level (FGL), and glycated hemoglobin (HbA1c)). The remainder of the serum material, after it had been centrifuged (10 min and approximately 2000 rpm), and the supernatant was removed, was transferred to the tubes and stored at −80 °C. TC, LDL-ch, HDL-ch, TG, and FGL were assayed using an enzymatic colorimetric method on an Alinity c analyzer (Abbott Laboratories, Lake Bluff, IL, USA). Glycated hemoglobin (HbA1c) was measured by the ion-exchange high-performance liquid-chromatography method using a D-10^TM^ Dual Program Reorder Pack (Bio-Rad, Hercules, CA, USA). Total Antioxidant Status (TAS) was determined using a reagent kit for the spectrophotometric method (Randox Laboratories, Crumlin, County Antrim, UK). This parameter informs about the amount of antioxidants in a sample and is related to the fact that ABTS (2,2’-Azino-di-[3-ethylbenzothiazoline sulfonate]), incubated with peroxidase (metmyoglobin) and H_2_O_2_, results in the formation of the radical cation ABTS^+^, which has a relatively stable blue-green coloration that can be measured at 600 nm. The presence of antioxidants in a sample causes suppression of dye production, which is proportional to their concentration. Units are expressed in an mmol Trolox equivalent/L, used as a standard to calculate antioxidant levels in the samples [12]. Control of the accuracy of the applied methods of determination was verified on the certified reference materials dedicated to each set. In both groups, all determinations were made, except for: FGL (T1DM group) and HbA1c (control group). The eGDR (estimated glucose-disposal rate) index takes into account waist circumference (WC), HbA1c, and the presence of hypertension (HT). Its formula is as follows: 21.158 − (0.090 × WC) − (3.407 × HT) − (0.551 × HbA1c). The value of WC is expressed in cm, HT as a dichotomous value (1 if present), and HbA1c in %. As eGDR decreases, insulin resistance (IR) increases [13]. It shows a strong correlation with IR based on euglycemic-hyperinsulinemic clamp and is associated with the occurrence of diabetic complications [13,14]. Our cut-off point was 8 mg/kg/min, selected on the basis of validation in adolescents with T1DM (76% sensitivity and 92% specificity) [15].

### 2.3. Nutritional Status and Nutrients Intake

Our assessment of the subjects’ nutritional status was based on anthropometric measurements (height, body weight, and circumference of the waist and hips). An analysis of the body composition was carried out by means of the bioelectric impedance method, using the professional medical analyzer Inbody 720 (Inbody, Cerritos, CA, USA). A detailed description of the measurement procedure and the devices used was extensively described in the previous study [4]. Blood pressure (BP) was measured at the beginning of the visit with a validated arm-pressure device, based on the oscillometric technique. Each participant was individually interviewed and asked to respond to a detailed 24 h nutritional intake survey about the day before their arrival at the clinic (T1DM group) or the Department of Bromatology (control group). The subjects were then given a food diary to record the food they ate for the next two days after the test (control group) or after leaving the hospital (T1DM group). Additionally, they were asked to complete a dietary questionnaire that included questions about the frequency of consumption of selected food groups [16]. The children and parents had been instructed on how to complete the document. In order to avoid discrepancies in the conducted interviews as well as in the anthropometric measurements, the examination was performed by the same dietitian. The “Dieta 6” program, which uses Polish databases of nutritional values of food products, was used to assess the consumption of nutrients from the diet. The obtained values were compared to the Polish nutritional standards for healthy children and adolescents [17], and in the case of the diabetics, according to Diabetes Poland guidelines [18] and the guidelines of the International Society for Pediatric and Adolescent Diabetes (ISPAD) [19]. The nutritional standards used in the publication are presented in Table 1.

### 2.4. Metabolic Syndrome Diagnosis

**Figure 2:**
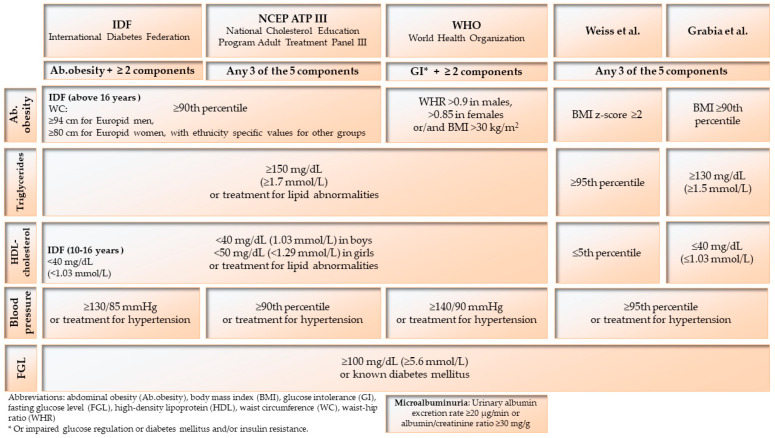
Criteria of metabolic syndrome (MetS) diagnosis in children and adolescents [7,8,9,14].

Each person was diagnosed for MetS using the four main criteria (Figure 2) proposed by the IDF, ATP, WHO, and Weiss et al. (modified) [7,8,9,20]. The MetS+ group included respondents who met at least one of the above-mentioned criteria. The rest of the diabetics were classified as the MetS− group. Standards for the pediatric population were used. Polish percentile grids were employed to evaluate the parameters expressed in percentiles [21,22]. For the purposes of this research, we modified the guidelines of Weiss et al., due to the lack of percentile grids for HDL-ch and TG [23].

### 2.5. Statistical Analysis

Statistical analysis of the results was performed with Statistica software (version 13 PL; TIBCO Software Inc., Palo Alto, CA, USA). Normal distribution of the variables was tested using the Shapiro–Wilk, Kolmogorov–Smirnov, and Lilliefors tests. Due to abnormal distribution, the data were presented in the form of medians and quartile ranges. In the case of quantitative variables, the Mann–Whitney U and Kruskal–Wallis ANOVA tests with post-hoc analysis were conducted. To assess the significance of the relationships between the qualitative variables, the chi-squared test of independence was used. If necessary, an additional V-square test and Yates correction were applied. Multiple correspondence analysis (MCA) is one of the exploratory statistical techniques that was used to detect all the common characteristics among individuals with T1DM who had MetS. The analyzed data were presented in a Burt’s matrix. Then, to determine the number of dimensions that the search space should have, a scree plot was used. *p* values < 0.05 were considered statistically significant. The study meets the assumptions of the minimum required sample size, assuming the maximum error value (10%) with a set confidence level (95%), which was calculated during study design, where we were guided by data on the estimated number of children and adolescents with T1DM in Poland [24].

## 3. Results

### 3.1. Study Characteristic

The characteristics of the population studied, along with information on the disease, type of insulin therapy, and the use of modern techniques of glycemic monitoring (GM) are presented in Table 2.

### 3.2. Prevalence of Metabolic Syndrome

Table 3 shows the percentage of respondents who met the individual criteria for the diagnosis of MetS. The syndrome was found in 33% of the diabetics—21% of the boys and 48% of the girls (some patients met more than one criterion), including 65%—CSII, 35%—MDI, and 30%—FGM or CGM; the remaining individuals (70%) did not use any modern GM support. In the healthy group, only 8% (*n* = 5) of adolescents had MetS. They were excluded from further comparative analysis.

Table 4 shows the parameters that have an impact on increased cardiovascular risk and some that are taken into account in the diagnosis of MetS. These results were also compared to those of a control group of healthy peers. Statistically significant differences in the medians of WHtR, BMI, HbA1c, eGDR, LDL-ch, HDL-ch, TG, and DBP were demonstrated.

**Figure 3:**
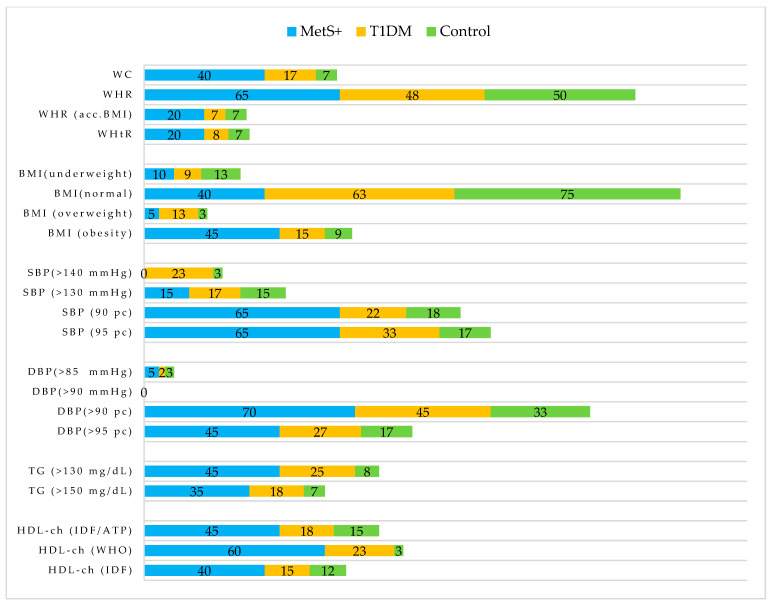
Percentage of participants meeting the metabolic syndrome components. Values are expressed as percentage of respondents (%). WHR (acc.BMI) includes the percentage of persons who are overweight or obese and, additionally, have a WHR above normal. BMI classifications for underweight, overweight, and obesity in the pediatric population correspond to the 10th, 85th, and 97th percentiles, respectively. Abbreviations: National Cholesterol Education Program Adult Treatment Panel III (ATP), body mass index (BMI), diastolic blood pressure (DBP), high-density lipoprotein cholesterol (HDL-ch), International Diabetes Federation (IDF), metabolic syndrome (MetS), systolic blood pressure (SBP), type 1 diabetes mellitus (T1DM), triglycerides (TG), waist circumference (WC), World Health Organization (WHO), waist–hip ratio (WHR), waist-to-height ratio (WHtR).

The parameters that most frequently exceeded the norm were those related to dyslipidaemia (Figure 3). However, a noteworthy observation is the large disproportion between the percentages of persons above normal SBP or DBP by numerical values and in terms of percentiles, e.g., 5% of patients with elevated DBP (in mmHg), as well as 70% and 45% of patients with DBP, were above the 90th and 95th percentiles, respectively.

It was observed that MetS+ patients had statistically significantly higher HbA1c values than those without MetS (8.9% vs. 6.9%, *p* < 0.01) (Table 5). MetS was found in almost half of the respondents, with values above 7%. Additionally, the concentration of TAS was measured and was statistically significantly lower in patients with MetS than in healthy volunteers (1.249 mmol/L vs. 1.578 mmol/L, *p* < 0.001).

### 3.3. Nutritional Status

Comparative analysis of body composition parameters (Table 6) showed statistically significant (*p* < 0.001) differences in the medians of percent body fat (PBF) and visceral fat area (VFA) between both the control and the MetS− groupsand MetS+ group.

### 3.4. Nutrients Intake

In comparison with the diets of their healthy peers, the diets of persons with T1DM and MetS+ (Table 7) were higher in saturated fatty acids (SFA: 17.6 g vs. 16.0 g, *p* < 0.01) and lower in products rich in mono- and polyunsaturated fatty acids (MUFA, PUFA): oleic acid (12.3 g vs. 21.4 g, *p* < 0.001), ω-3 (0.831 g vs. 1.3 g, *p* < 0.001), and ω-6 (4.9 g vs. 7.6 g, *p* < 0.001), as well as dietary fiber (18.1 g vs. 19.6 g). Although healthy volunteers declared high total fat intakes (28.1 g), the majority of the fats they consumed came from MUFA (24.6 g) and PUFA (9.8 g), not SFA (16.0 g). The MetS+ group also proved to have higher saccharose intakes than MetS− subjects (39.5 g vs. 33 g).

It was shown that, compared to persons with normal values, volunteers who had lower HDL-ch levels not only consumed large amounts of SFA relative to total daily energy expenditure (TDEE) (8.4% vs. 8.2%TDEE) but also reported statistically significantly lower consumption of MUFA (7.6% vs. 10% TDEE, *p* < 0.05), oleic acid (13.1 g vs. 16.6 g, *p* < 0.05), LA (4.7 g vs. 6.2 g, *p* < 0.05), anddietary fiber (17.4 g vs. 18.7 g, *p* < 0.05). Additionally, differences were observed in the intake of PUFA (6.2 g vs. 7.8 g) from the ω-3 family: ALA (626 mg vs. 824 mg), EPA + DHA (57 mg vs. 71 mg), and ω-6 (4.8 g vs. 6.2 g). Subjects with higher TG levels consumed statistically significantly more SFA (9.1% vs. 8.1% TDEE, *p* < 0.05) but less oleic acid (13.5 g vs. 16.6 g, *p* < 0.05). As well, differences were noted in the intake of PUFA (6.4 g vs. 7.7 g), EPA + DHA (60 mg vs. 71 mg), LA (5.6 g vs. 6.1 g), and dietary fiber (16.9 g vs. 18.9 g). Participants with high BP consumed high amounts of salt (8.6 g vs. 9.64 g, *p* < 0.05) but lower quantities of oleic acid (15 g vs. 16.5 g) and EPA + DHA (57 mg vs. 71 mg).

Table 8 shows the percentages of subjects who met the nutritional standards for selected nutrients. The vast majority of participants met the standard for basic nutrients, such as protein, fat, or carbohydrates. A statistically significant relationship was observed between the control group and MetS+ in the distribution of the percentage of nutritional norms for MUFA and LA (*p* < 0.001) as well asALA (*p* < 0.01). It was found that every third diabetic with MetS consumed more than 10% TDEE of SFA, and more than 90% and 75% of the patients failed to meet PUFA and MUFA recommendations, respectively.

### 3.5. Nutritional Habits

Compared to their healthy peers (Table 9), members of the MetS+ group chose wheat bread (90% vs. 78%) more frequently (from several times a week to several times a day) than wholemeal bread (45% vs. 73%). They were also more likely to eat potatoes (100% vs. 56%) than groats and pastas (40% vs. 53%). Over 35% consumed fast-food products at least once to several times a week, and 80% ate fried foods. It has been observed that only 60% chose white meat and more than 90% ate red meat at least once or several times a week. Only 50% ate vegetables at least once a day, while 65% ate fruit. As many as 60% drank products containing sweeteners once or several times a week.

### 3.6. Insulin Therapy and Modern Glucose-Monitoring Systems

Comparative analyses of obtained results of cardiovascular biomarkers, body composition analysis, antioxidant status, metabolic management, and dietary nutrient intake in relation to insulin therapy and modern glycemic-monitoring systems are included in the Supplementary materials (Supplementary Tables S1–S5). It was shown that the largest subgroup of patients with MetS were participants who used CSII without the support of modern GM. Their HbA1c (8.3%), eGDR (8.7 mg/kg/min), and TAS (1.099 mmol/L) were worse than the levels of these indicators in those MetS patients who used CSII and CGM (7.9%, 6.5 mg/kg/min, 1.259 mmol/L). Similar observations were noted in the MDI group (Tables S1 and S2). Based on Table S3, it was found that there were statistically significant differences in median TG levels between insulin therapies (CSII vs. MDI: 60 mg/dL vs. 99 mg/dL, *p* < 0.02) and between groups using FGM or CGM and those not using either system (FGM/CGM vs. no GM: 63/54 mg/dL vs. 80 mg/dL, *p* < 0.05). It was also noted that participants supported with any of the systems had a statistically significantly lower HbA1c than those who did not use any modern support (6.8%/6.7% vs. 8.1%, *p* < 0.001). Table S4 contains the results of the body-composition analysis, which showed that the CSII group had a lower VFA than MDI (46 cm^2^ vs. 52 cm^2^), which was also observed in the modern GM participants (FGM/CGM vs. no GM: 46 cm^2^/44 cm^2^ vs. 49 cm^2^).

### 3.7. Multiple Correspondence Analysis

**Figure 4:**
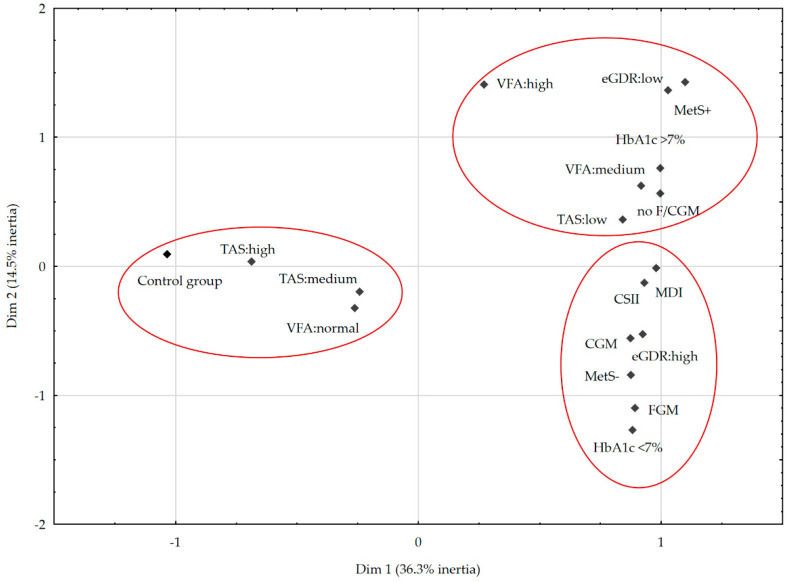
Multivariate correspondence analysis coordinate plot. Abbreviations: continuous glucose monitoring (CGM), continuous subcutaneous insulin infusion (CSII), dimension (dim), estimated glucose-disposal resistance (eGDR), flash glucose monitoring (FGM), glucose monitoring (GM), glycated hemoglobin (HbA1c), multiple daily injections (MDI), metabolic syndrome (MetS), total antioxidant status (TAS), visceral fat area (VFA).

MCA was used to identify the structure of associations between metabolic control, antioxidant status, visceral fat and the occurrence of MetS, taking into account insulin therapy and the usage of modern glycemic monitoring. A prepared scree plot suggested adoption of two-dimensional space for analysis. After determining the number of dimensions in the next step, the coordinates of the column profiles were calculated in the new orthonormal framework. Figure 4 shows the results of the MCA. The two-dimensional plot explains 51% of the total variability, which allows us to distinguish the following three groups:

(1) The first quadrant contained participants with MetS, characterized by poor metabolic management (HbA1c > 7%), low eGDR (<8 mg/kg/min), low TAS (<1.3 mmol/L), and medium (>50 cm^2^) to high (>100 cm^2^) VFA, was not supported by FGM or CGM.
(2) The opposite (III) and side quadrant (II) included healthy peers with moderate (1.3–1.8 mmol/L) to high (>1.8 mmol/L) TAS and normal VFA (<50 cm^2^).
(3) The last quadrant (IV) included individuals without MetS with optimal metabolic control (HbA1c < 7%) and high eGDR (>8 mg/kg/min), who were using CSII or MDI and FGM or CGM.

## 4. Discussion

A previously published systematic review [10] showed that nearly 30% of diabetics were diagnosed with metabolic syndrome. Our study confirmed this: 33% of the diabetics had MetS. However, this was the case among girls (48%) more often than boys (21%), which has also been noted by other authors, e.g., Köken et al. (12% vs. 10%), Soliman et al. (18% vs. 8%), Szadkowska et al. (11% vs. 7%), and Valerio et al. (16% vs. 4%). In our study, most diagnoses of MetS were found based on the ATP criteria (25%), which is consistent with the literature data: 14% [25], 30% [26], and the lowest according to the IDF (8%), as well as in other authors from 3% [27] up to 13% [28], with the exception of the furthest outlier, 24% [29]. This is due to the different intensity of the components included in the criteria—the ATP allows for selecting three components out of five, while the IDF places diagnosed abdominal obesity as the first one, ahead of the other components. The second fundamental difference regards the values of the cut-off points used—mainly numerical values, rarely percentiles. When national percentile grids were used (according to the modified criteria of Weiss et al.), the percentage of MetS diagnoses fell to 13%. Another interesting thing is that when we used cut-off points from the percentile grids for SBP and DBP from the rarely recognized elevated BP (above 130/85 mmHg) and almost-never recognized high BP (above 140/90 mmHg), these percentages as much as doubled. In the case of the HDL-ch parameter, the percentage of non-compliant persons had a considerable discrepancy: 40%, 45%, and 60% according to the criteria of the IDF, ATP, and WHO, respectively. Therefore, it would be worthwhile to consider modifying the numerical values of some MetS components to those related to national percentile grids.

Due to the production of large amounts of oxygen free radicals and/or reduced antioxidant defense, oxidative stress has a significant impact on the development of insulin resistance and most diabetic complications [30]. Long-term high glycemia promotes overproduction of oxygen free radicals. In the presence of low antioxidant activity, beta cells are more susceptible to the adverse effects of oxidative stress and their destruction is exacerbated [31]. In our study, subjects with T1DM as well as with MetS had statistically significantly lower TAS values than the control group. Similar results were obtained by other authors [32,33].

Our investigation showed that MetS subjects had a statistically significantly higher VFA compared to the MetS− group. The same was seen in patients with MDI and CSII, as well as non-users of modern GM systems and those supporting themselves with FGM or CGM. Many studies confirmed that individuals with high VFA considerable risk of developing cardiometabolic complications and a number of related diseases [34]. Therefore, it is particularly threatening for individuals with T1DM.

Our study proved that MetS patients had much lower eGDR levels, which could be related to the occurrence of high IR. Similar results were observed by Köken et al. [15]. Moreover, during our analysis in different subgroups, the index increased depending on the insulin therapy and modern GM systems used. In combination with its high specificity and negative predictive value [15] for excluding the diagnosis of MetS, it should be considered as one of the components of MetS. An additional advantage is that it can be calculated quickly using the results obtained during routine follow ups, and its numerous correlations make it easily applicable in clinical practice.

It was shown that the average BMI value in young diabetics without MetS (19.8 kg/m^2^) did not differ from the results reported by our previous study (19.2 kg/m^2^) [4] or other authors conducting research in similar age groups—19.5 kg/m^2^ [35], 21.3 kg/m^2^ [36] and 21.5 kg/m^2^ [37,38]. The percentage of fat mass in our study was 16%, while the other authors reported different outcomes—18.5% [35], 19.1% [4], 21.9% [38] and 22.4% [37].

Lifestyle medicine is a key element in the prevention and treatment of metabolic disorders. The most important role is played by modification of eating habits and physical activity [10,37,38].

Diabetics without MetS consumed protein at 18.1% TDEE. Similar results were obtained by other authors: 16.9% TDEE [39] and 16% TDEE [40,41]. Regarding consumption of SFA (16.4 g, 8.8% TDEE), MUFA (14.2 g, 7.5% TDEE), and PUFA (6.2 g, 10.3% TDEE), our results differed from those reported by Katz et al. (SFA 12.4% TDEE) [41] and Thomson et al. (37.4 g, 33.2 g, and 11.2 g, respectively) [42].

Our study found disturbing results suggesting inadequate nutrient intakes in patients with abnormal HDL-ch, TG, and BP levels. Participants with low HDL-ch levels consumed high amounts of SFA (8.4% TDEE) but low quantities of MUFA (7.6% TDEE), EPA + DHA (57 mg), and LA (4.7 g), which had the strongest influence in this case. We observed similar results in patients with high TG levels, who had a lot of products rich in SFA (9.1% TDEE) in their diet, and too few foods that were high in EPA + DHA (60 mg) and LA (5.6 g). Patients with high BP consumed large amounts of salt (8.4 g) and low amounts of oleic acid (15 g) and EPA + DHA (57 mg). An adequate quantity of HDL-ch has a beneficial anti-atherosclerotic effect, which is related primarily to its participation in cholesterol re-transport. It has been shown that its concentration is increased by 0.4 mg/dL for each kilogram of body-weight loss and by 6 mg/dL as a consequence of moderate-intensity physical activity (approx. 300 min a week) [43]. However, the best results can be achieved by reducing trans fats and carbohydrates in the diet in favor of unsaturated FA [44]. If a nutritional intervention is aimed at lowering TG, a significant role is played by the reduction in body weight and the consumption of simple carbohydrates as well as by replacing SFA with PUFA and introducing regular physical activity. This improves tissue insulin sensitivity, which influences the TG level [44,45,46]. The consumption of ω-3 FA (approx. 2–4 g/day) favors the reduction in TG by about 25–30%, but also, importantly, has a beneficial effect on inflammatory markers [47]. The inclusion of products with a low glycemic index and load in the diet efficiently lowers the concentration of TG; then, such foods that have a low plasma glucose-absorption profile allow for its gradual release during intestinal transit [48]. Non-pharmacological treatment of arterial hypertension should include: normalization of body weight, reduction in SFA and salt intake, increased consumption of vegetables and fruit, and systematic physical activity [49]. A meta-analysis by Aburto et al. showed that reducing sodium intake causes a decrease of 3.4/1.5 mmHg in SBP/DBP [50]. By monitoring its consumption, it is also possible to reduce the number and doses of antihypertensive drugs as well as the risk of cardiovascular events [49].

Lifestyle changes should be promoted in all patient groups and must become an integral part of the treatment of metabolic disorders.

Our study has several strengths and weaknesses. Firstly, some of the nutrient intake data were retrospectively collected, which could have influenced the results by underestimating or overestimating these parameters. Secondly, the size of the groups is not too large. However, compared to other studies, this group of patients maintained an appropriate test power, at about 90%. However, an extremely large advantage of the present study over others is the very extensive and comprehensive screening of participants in terms of the various criteria of MetS definition, cardiovascular biomarkers, nutrients intake, eating habits, and nutritional status, including a comparison of the type of insulin therapy and modern GM used. An additional advantage is the inclusion of a control group of healthy children, which enabled comparative analysis.

## 5. Conclusions

The study found that a high percentage of young diabetics had MetS. These participants displayed many inappropriate eating behaviors (meaning a diet low in mono- and polyunsaturated fatty acids and rich in saturated fatty acids). This long-term presence in combination with an excessive content of fat tissue, especially visceral, as well as incorrect results of laboratory tests (cardiovascular biomarkers) and confirmed low antioxidant status, may result in difficulty in maintaining metabolic control, which, in turn, may lead to faster development of diabetic complications.

## Figures and Tables

**Table 1:** Nutritional standards for children and adolescents.

Nutrients	Polish Standards for Healthy Children [17]	Diabetes Poland Standards [18]	ISPAD Standards [19]
Protein	10–20%	15–20%	15–20%
Carbohydrates	45–65%	45% (up to 60% if low-GI, high-fiber food)	45–50%
Fat	20–35%	25–40%	up to 30–35%
SFA	as low as possible	<10%	<10%
MUFA	-	<20%	-
PUFA	-	6–10%	-
EPA + DHA	250 mg	-	-
ALA	0.5%	-	-
LA	4%	-	-
Dietary fiber	19 g (10–15 y), 21 g (16–18 y)	>25 g or 15 g/1000 kcal	Age (y) + 5 g

Percentage values are expressed as total daily energy intake. Abbreviations: alpha-linolenic acid (ALA), docosahexaenoic acid (DHA), eicosapentaenoic acid (EPA), glycemic index (GI), International Society for Pediatric and Adolescent Diabetes (ISPAD), linoleic acid (LA), monounsaturated fatty acids (MUFA), polyunsaturated fatty acids (PUFA), saturated fatty acids (SFA), years (y).

**Table 2:** Characteristics of the study cohort.

Parameter	Participants	
	with T1DM (*n* = 60)	without T1DM (*n* = 60)
	Me ± IQR	
Age (years)	14 (12–16)	15 (13–16)
Body height (cm)	166 (156–173)	168 (162–176)
Body weight (kg)	54 (45–66)	58 (47–69)
Age of diagnosis (years)	9 (7–11)	
Diabetes duration (years)	5 (2–7)	
HbA1c (%)	7.6 (6.6–10.2)	
	*n* (%)	
Gender (girls/boys)	27 (45%)/33 (55%)	16 (27%)/44 (73%)
Type of insulin therapy (MDI/CSII)	23 (38%)/37 (52%)	
Type of glucose-monitoring system (FGM/CGM)	18 (70%)/8 (30%)	

Values are expressed as median and interquartile range (Me (Q_1_–Q_3_) or number and percentage of respondents (*n* (%)). Abbreviations: continuous glucose monitoring (CGM), continuous subcutaneous insulin infusion (CSII), flash glucose monitoring (FGM), multiple daily injections (MDI), type 1 diabetes mellitus (T1DM).

**Table 3:** Prevalence of metabolic syndrome, based on various criteria.

Criteria	T1DM Group (*n* = 60)		Patients without T1DM (*n* = 60)	
	Total	Girls/Boys	Total	Girls/Boys
ATP	15 (25%)	11 (41%)/4 (12%)	3 (5%)	1 (6%)/2 (5%)
IDF	5 (8%)	3 (11%)/2 (6%)	3 (5%)	0/3 (7%)
WHO	8 (13%)	4 (15%)/4 (12%)	1 (2%)	0/1 (2%)
Grabia et al. (modified Weiss et al.)	11 (18%)	9 (33%)/2 (6%)	1 (2%)	0/1 (2%)

Values are expressed as a number and percentage of respondents (*n* (%)). Abbreviations: National Cholesterol Education Program Adult Treatment Panel III (ATP), International Diabetes Federation (IDF), type 1 diabetes mellitus (T1DM), World Health Organization (WHO).

**Table 4:** Comparison of cardiovascular biomarkers.

Parameter	MetS+ (*n* = 20)	MetS− (*n* = 40)	Control Group (*n* = 55)	*p*-Value	
	Me ± IQR			MetS+ vs. MetS−	MetS+ vs. Control
WC (cm)	73 (69–78)	66 (62–70)	70 (67–74)	<0.001	N/S
WHR	0.87 (0.84–0.9)	0.87 (0.81–0.91)	0.88 (0.85–0.92)	N/S	N/S
WHtR	0.40 (0.39–0.42)	0.40 (0.38–0.42)	0.44 (0.42–0.48)	<0.001	<0.001
BMI (kg/m^2^)	22.6 (19.9–24.5)	19.8 (17.9–21.2)	20.3 (18.6–22.0)	<0.01	<0.5
FGL (mg/dL)	-	-	98 (93–103)	-	-
HbA1c (%)	8.9 (7.4–11.4)	6.9 (6.4–9.2)	-	<0.001	-
eGDR (mg/kg/min)	8.0 (6.3–10.0)	10.8 (8.8–11.6)	-	<0.001	-
TC (mg/dL)	157 (124–187)	148 (123–170)	143 (131–187)	N/S	N/S
LDL-ch (mg/dL)	102 (74–111)	80 (66–101)	86 (76–110)	<0.5	N/S
HDL-ch (mg/dL)	44.5 (34.5–57.5)	59 (48–71)	57 (52–64)	<0.001	<0.001
TG (mg/dL)	101 (67–143)	60 (47–91)	59 (45–76)	<0.001	<0.001
SBP (mmHg)	120 (110–128)	114 (109–118)	118 (110–125)	<0.5	N/S
DBP (mmHg)	74 (70–80)	70 (66–73)	70 (65–74)	<0.01	<0.5

Values are expressed as median and interquartile range (Me (Q_1_–Q_3_). Statistically significant differences between the medians were detected by the Mann–Whitney U test. Abbreviations: National Cholesterol Education Program Adult Treatment Panel III (ATP), body mass index (BMI), diastolic blood pressure (DBP), estimated glucose disposal resistance (eGDR), fasting glucose level (FGL), high-density lipoprotein cholesterol (HDL-ch), glycated hemoglobin (HbA1c), International Diabetes Federation (IDF), low-density lipoprotein cholesterol (LDL-ch), metabolic syndrome (MetS), systolic blood pressure (SBP), total cholesterol (TC), triglycerides (TG), waist circumference (WC), waist–hip ratio (WHR), waist-to-height ratio (WHtR), World Health Organization (WHO).

**Table 5:** HbA1c and TAS values, depending on metabolic control.

Study Group	HbA1c (%)	TAS (mmol/L)	*p*-Value	HbA1c Group	HbA1c	*p*-Value	HbA1c (%)	*p*-Value	TAS (mmol/L)	*p*-Value
	Me ± IQR				*n*(%)		Me ± IQR		Me ± IQR	
MetS+ (*n* = 20)	8.9 (7.4–11.4)	1.249 (1.054–1.322)	MetS+ vs. MetS− <0.01^HbA1c, TAS^	**(<7%)**	3 (12%)	<0.01	6.7 (6.2–7.0)	<0.001	1.230 (1.212–1.382)	MetS+^HbA1c>7%^ vs. MetS−^HbA1c>7%^ <0.01
				**(>7%)**	17 (49%)		9.9 (7.7–12.2)		1.243 (1.041–1.314)	
MetS− (*n* = 40)	6.9 (6.4–9.2)	1.394 (1.225–1.595)	MetS+ vs. Control MetS− vs. Control <0.001^TAS^	**(<7%)**	22 (88%)		6.4 (6.0–6.7)	<0.001	1.403 (1.206–1.533)	
				**(>7%)**	18 (51%)		9.7 (7.8–12.1)		1.370 (1.256–1.655)	
Control group (*n* = 55)	-	1.579 (1.457–1.799)		-						

Values are expressed as median and interquartile range (Me (Q_1_–Q_3_) or number and percentage of respondents (*n* (%)). Statistically significant differences between the medians or percentages were detected by the Mann–Whitney U or the chi-squared test, respectively. Abbreviations: glycated hemoglobin (HbA1c), metabolic syndrome (MetS), total antioxidant status (TAS).

**Table 6:** Comparison of body-composition analysis parameters.

Parameter	MetS+ (*n* = 20)	MetS− (*n* = 40)	Control Group (*n* = 55)	*p*-Value	
	Me ± IQR			MetS+ vs. MetS−	MetS+ vs. Control
Body weight (kg)	64 (52–73)	52 (43–59)	58 (48–68)	<0.5	NS
Body height (cm)	167 (156–173)	164 (155–173)	169 (163–176)	NS	NS
TBW (L)	33 (28–38)	30 (26–38)	37 (31–41)	NS	NS
SMM (kg)	24 (21–29)	22 (19–29)	24 (18–30)	NS	NS
Protein (kg)	8.6 (7.6–10.2)	8.1 (6.9–10.2)	9.1 (7.9–10.5)	NS	NS
Minerals (kg)	3.3 (2.7–3.6)	2.9 (2.5–3.5)	3.2 (2.8–3.9)	NS	NS
PBF (%)	26 (21–33)	16 (12–23)	14 (12–16)	<0.001	<0.001
VFA (cm^2^)	77 (54–100)	42 (28–48)	35 (26–44)	<0.001	<0.001

Values are expressed as median and interquartile range (Me (Q_1_–Q_3_). Statistically significant differences between the medians were detected by the Mann–Whitney U test. Abbreviations: metabolic syndrome (MetS), percentage body fat (PBF), skeletal muscle mass (SMM), total body water (TBW), visceral fat area (VFA).

**Table 7:** Consumption of selected nutrients with the diet.

Nutrient	MetS+ (*n* = 20)	MetS− (*n* = 40)	Control Group (*n* = 55)	*p*-Value (MetS+ vs. Control)
	Me ± IQR			
Main nutrients				
Energy (kcal)	1760 (1697–1924)	1803 (1574–1916)	1859 (1735–1935)	N/S
Protein (%TDEE)	20.0 (16.4–20.8)	18.1 (15.8–20.6)	16.4 (13.1–18.6)	<0.01
Carbohydrate (%TDEE)	56.5 (50.8–59.9)	54.8 (50.6–59.5)	55.4 (51.5–61.1)	N/S
Fat (%TDEE)	22.8 (20.0–28.1)	24.3 (21.2–29.1)	28.1 (23.7–32.4)	<0.01
Fatty acids				
SFA (g)	17.6 (14.3–20.7)	16.4 (15.3–17.5)	16.0 (15.1–17.9)	N/S
Palmitic acid (g)	10.4 (9.4–11.4)	10.1 (8.9–11.6)	9.8(8.6–10.8)	N/S
MUFA (g)	14.2 (11.9–17.7)	14.2 (11.2–19.2)	24.6 (20.5–28.5)	<0.001
Oleic acid (g)	12.3 (10.6–14.8)	13.1 (10.8–16.3)	21.4 (16.6–25.4)	<0.001
PUFA (g)	5.8 (5.0–7.1)	6.2 (4.6–8.1)	9.8 (7.4–11.6)	<0.001
LC-PUFA (g)	0.069 (0.036–0.205)	0.069 (0.04–0.093)	0.093 (0.06–0.231)	<0.05
ω-3 (g)	0.831 (0.569–1.178)	0.688 (0.554–1.28)	1.3 (0.948–1.6)	<0.001
ALA (g)	0.688 (0.524–0.817)	0.554 (0.478–0.879)	1.2 (0.822–1.4)	<0.001
EPA (g)	0.014 (0.006–0.045)	0.012 (0.007–0.017)	0.024 (0.008–0.052)	N/S
DHA (g)	0.039 (0.024–0.148)	0.038 (0.022–0.066)	0.066 (0.038–0.137)	<0.001
ω-6 (g)	4.9 (4.2–5.9)	5.5 (3.9–6.5)	7.6 (6.1–9.6)	<0.001
LA (g)	4.8 (4.1–5.9)	5.5 (3.8–6.4)	7.3 (6.0–9.4)	<0.001
AA (g)	0.048 (0.032–0.151)	0.048 (0.031–0.096)	0.111 (0.073–0.181)	<0.05
Carbohydrates				
Glucose (g)	8.1 (4.0–9.6)	4.8 (2.6–6.5)	6.8 (5.1–8.1)	N/S
Fructose (g)	10.1 (4.6–12.9)	7.3 (4.2–11.1)	8.3 (6.5–11.0)	N/S
Saccharose (g)	39.5 (30.2–54.8)	33.1 (13.7–45.0)	44.1 (35.1–51.6)	N/S
Dietary fiber (g)	18.1 (16.6–21.4)	18.0 (13.8–20.8)	19.6 (16.0–23.0)	N/S

Values are expressed as median and interquartile range (Me (Q_1_–Q_3_). Statistically significant differences between the medians were detected by the Mann–Whitney U test. Abbreviations: arachidonic acid (AA), alpha-linolenic acid (ALA), docosahexaenoic acid (DHA), eicosapentaenoic acid (EPA), fatty acids (FA), linoleic acid (LA), long-chain polyunsaturated fatty acids (LC-PUFA), metabolic syndrome (MetS), monounsaturated fatty acids (MUFA), polyunsaturated fatty acids (PUFA), saturated fatty acids (SFA), total daily energy expenditure (TDEE).

**Table 8:** Implementation of nutritional standards for selected nutrients.

Nutrient	Recommendation	MetS+ (*n* = 20)	MetS− (*n* = 40)	Control Group (*n* = 55)	*p*-Value (MetS+ vs. Control)
Main nutrients					
Protein	<10%	0 (0%)	0 (0%)	3 (5%)	<0.05
	10–20%	15 (75%)	33 (83%)	49 (90%)	
	>20%	5 (25%)	7 (17%)	3 (5%)	
Protein ^PolDiab^	<15%	2 (10%)	6 (15%)	21 (38%)	<0.01
	15–20%	13 (65%)	27 (68%)	31 (56%)	
	>20%	5 (25%)	7 (17%)	3 (6%)	
Fat	<20%	5 (25%)	6 (15%)	4 (7%)	<0.05
	20–35%	15 (75%)	33 (83%)	47 (86%)	
	>35%	0 (0%)	1 (2%)	4 (7%)	
Fat ^PolDiab^	<25%	14 (70%)	23 (58%)	20 (36%)	<0.01
	25–40%	6 (30%)	17 (43%)	35 (64%)	
	>40%	0 (0%)	0 (0%)	0 (0%)	
Carbohydrates	<45%	0 (0%)	2 (5%)	2 (3%)	N/S
	45–65%	18 (90%)	37 (93%)	52 (95%)	
	>65%	2 (10%)	1 (2%)	1 (2%)	
Carbohydrates ^PolDiab^	<45%	0 (0%)	2 (5%)	2 (4%)	N/S
	45–60%	16 (80%)	31 (78%)	39 (71%)	
	>60%	4 (20%)	7 (17%)	14 (25%)	
Carbohydrates ^ISPAD^	<45%	0 (0%)	2 (5%)	2 (4%)	N/S
	45–50%	5 (25%)	9 (22%)	9 (16%)	
	>50%	15 (75%)	29 (73%)	44 (80%)	
Fatty acids					
SFA	<10%	13 (65%)	29 (73%)	49 (89%)	<0.05
	≥10%	7 (35%)	11 (27%)	6 (11%)	
MUFA	<10%	15 (75%)	29 (73%)	15 (27%)	<0.001
	10–20%	5 (25%)	11 (27%)	38 (69%)	
	>20%	0 (0%)	0 (0%)	2 (4%)	
LA	<3.5%	19 (95%)	31 (78%)	26 (47%)	<0.001
	3.5–4.5%	1 (5%)	7 (18%)	16 (29%)	
	>4.5%	0 (0%)	2 (4%)	13 (24%)	
ALA	≤0.5%	17 (85%)	33 (83%)	23 (42%)	<0.01
	>0.5%	3 (15%)	7 (17%)	32 (58%)	
EPA + DHA	≤250 mg	17 (85%)	37 (93%)	44 (80%)	N/S
	>250 mg	3 (15%)	7 (3%)	11 (20%)	
PUFA	<6%	18 (90%)	39 (97%)	45 (82%)	N/S
	6–10%	2 (10%)	1 (2%)	9 (16%)	
	>10%	0 (0%)	0 (0%)	1 (2%)	
Carbohydrates					
Saccharose	≤10%	12 (60%)	30 (75%)	30 (55%)	N/S
	>10%	8 (40%)	10 (25%)	25 (45%)	
Dietary fiber	>19 g	13 (65%)	27 (68%)	26 (47%)	N/S
	≥19 g	7 (35%)	13 (32%)	29 (53%)	
Dietary fiber ^PolDiab^	<25 g	18 (90%)	39 (98%)	46 (83%)	N/S
	≥25 g	2 (10%)	1 (2%)	9 (17%)	

The standards used refer to the Polish guidelines for the general population [17], unless there are separate recommendations for patients with diabetes mellitus (PolDiab—Polish guidelines for diabetics [18], ISPAD—international guidelines for young diabetics [19]). Values are expressed as number and percentage of respondents (*n* (%)). Statistically significant relationships between the numbers were detected by the chi-squared test. The data in the “Recommendation” column refer to the percentage of total daily energy expenditure. Abbreviations: alpha-linolenic acid (ALA), docosahexaenoic acid (DHA), eicosapentaenoic acid (EPA), linoleic acid (LA), metabolic syndrome (MetS), monounsaturated fatty acids (MUFA), polyunsaturated fatty acids (PUFA), saturated fatty acids (SFA).

**Table 9:** Frequency of consumption of selected groups of food products.

Food Products		Never	1–3 Times a Month	Once a Week	Several Times a Week	Once a Day	Several Times a Day
Wheat bread	MetS+	0 (0%)	1 (5%)	1 (5%)	5 (25%)	5 (25%)	8 (40%)
	MetS−	2 (5%)	2 (5%)	3 (8%)	8 (20%)	6 (15%)	19 (47%)
	Control	6 (11%)	2 (4%)	4 (7%)	21 (38%)	15 (27%)	7 (13%)
Wholemeal bread	MetS+	2 (10%)	3 (15%)	6 (30%)	5 (25%)	1 (5%)	3 (15%)
	MetS−	3 (8%)	2 (5%)	8 (20%)	11 (27%)	10 (25%)	6 (15%)
	Control	6 (11%)	3 (5%)	6 (11%)	25 (45%)	12 (22%)	3 (6%)
Groats, pasta, rice	MetS+	0 (0%)	2 (10%)	10 (50%)	7 (35%)	1 (5%)	0 (0%)
	MetS−	0 (0%)	7 (17%)	12 (30%)	20 (50%)	1 (3%)	0 (0%)
	Control	0 (0%)	8 (15%)	9 (16%)	35 (63%)	2 (4%)	1 (2%)
Potatoes	MetS+	0 (0%)	0 (0%)	0 (0%)	9 (45%)	11 (55%)	0 (0%)
	MetS−	0 (0%)	2 (5%)	2 (5%)	21 (52%)	15 (38%)	0 (0%)
	Control	0 (0%)	11 (20%)	13 (23%)	25 (46%)	4 (7%)	2 (4%)
Red meat	MetS+	1 (5%)	7 (35%)	6 (30%)	6 (30%)	0 (0%)	0 (0%)
	MetS−	6 (15%)	6 (15%)	5 (12%)	19 (48%)	2 (5%)	2 (5%)
	Control	3 (5%)	5 (9%)	15 (27%)	30 (55%)	2 (4%)	0 (0%)
White meat	MetS+	0 (0%)	2 (10%)	6 (30%)	11 (55%)	1 (5%)	0 (0%)
	MetS−	1 (2%)	3 (7%)	5 (13%)	27 (68%)	2 (5%)	2 (5%)
	Control	0 (0%)	2 (4%)	7 (13%)	43 (78%)	3 (5%)	0 (0%)
Fried products	MetS+	1 (5%)	3 (15%)	6 (30%)	5 (25%)	5 (25%)	0 (0%)
	MetS−	1 (3%)	4 (10%)	16 (40%)	18 (44%)	0 (0%)	1 (3%)
	Control	1 (2%)	6 (11%)	14 (25%)	30 (55%)	4 (7%)	0 (0%)
Fast-food	MetS+	2 (10%)	11 (55%)	5 (25%)	2 (10%)	0 (0%)	0 (0%)
	MetS−	4 (10%)	25 (63%)	10 (25%)	1 (2%)	0 (0%)	0 (0%)
	Control	9 (16%)	37 (67%)	8 (15%)	1 (2%)	0 (0%)	0 (0%)
Fruit	MetS+	0 (0%)	0 (0%)	1 (5%)	6 (30%)	9 (45%)	4 (20%)
	MetS−	0 (0%)	3 (8%)	0 (0%)	13 (32%)	11 (28%)	13 (32%)
	Control	0 (0%)	0 (0%)	2 (3%)	18 (33%)	17 (31%)	18 (33%)
Vegetables	MetS+	0 (0%)	0 (0%)	2 (10%)	8 (40%)	5 (25%)	5 (25%)
	MetS−	0 (0%)	0 (0%)	2 (5%)	13 (33%)	8 (20%)	17 (42%)
	Control	0 (0%)	0 (0%)	3 (6%)	17 (31%)	19 (35%)	16 (28%)
Beverages with sweeteners	MetS+	2 (10%)	3 (15%)	3 (15%)	10 (50%)	2 (10%)	0 (0%)
	MetS−	9 (22%)	4 (10%)	12 (30%)	9 (22%)	5 (13%)	1 (3%)
	Control	13 (24%)	8 (14%)	11 (20%)	12 (22%)	10 (18%)	1 (2%)
Energy drink	MetS+	13 (65%)	2 (10%)	4 (20%)	1 (5%)	0 (0%)	0 (0%)
	MetS−	30 (75%)	3 (7%)	6 (15%)	1 (3%)	0 (0%)	0 (0%)
	Control	47 (85%)	5 (9%)	3 (6%)	0 (0%)	0 (0%)	0 (0%)

Abbreviation: metabolic syndrome (MetS).

## Data Availability

The data presented in this study are available on request from the corresponding author.

## Supplementary Materials

The following supporting information can be downloaded at: https://www.mdpi.com/article/10.3390/nu14122435/s1; Table S1: HbA1c, eGDR, and TAS values depending on insulin therapy and modern GM systems used, Table S2: TAS, HbA1c, and eGDR values depending on insulin therapy and modern GM systems used, Table S3: Comparison of cardiovascular biomarkers depending on insulin therapy and modern GM systems used, Table S4: Comparison of body-composition analysis parameters depending on insulin therapy and modern GM systems used, Table S5: Consumption of the selected nutrients with diet depending on insulin therapy and modern GM systems used.
