# Supplementary material for: Prevalence of Metabolic Syndrome in Relation to Cardiovascular Biomarkers and Dietary Factors among Adolescents with Type 1 Diabetes Mellitus
Source: Nutrients. 2022 Jun 12;14(12):2435. doi: 10.3390/nu14122435 (PMC9228781; doi:10.3390/nu14122435)
Supplement: Supplementary file 1 [file nutrients-14-02435-s001.zip › nutrients-1762764-supplementary.pdf]

nutrients

MDPI

Supplementary Material

Prevalence of Metabolic Syndrome in Relation to Cardiovascular Biomarkers and Dietary Factors among Adolescents with Type 1 Diabetes Mellitus

Monika Grabia[1], Renata Markiewicz-Żukowska[1], Katarzyna Socha[1], Agnieszka Polkowska[2], Aneta Zasim[2], Karolina Boruch[3] and Artur Bossowski[2]

[1] Department of Bromatology, Faculty of Pharmacy with the Division of Laboratory Medicine, Medical University of Białystok, Mickiewicza 2D Street, 15-222 Białystok, Poland
[2] Clinic of Paediatrics, Endocrinology, Diabetology with Subdivision of Cardiology, Children's University Clinical Hospital in Białystok
[3] Clinic of Paediatrics, Rheumatology, Immunology and Bone Metabolic Diseases, Children's University Clinical Hospital in Białystok

Table S1. HbA1c, eGDR and TAS values depending on insulin therapy and modern GM systems used.

Group	Insulin therapy	GM systems	n (%)	HbA1c (%)	eGDR (mg/kg/min)	TAS (mmol/L)
MetS+	CSII	**Total**	**13**	**7.9 (7.6-9.9)**	**8.0 (6.5-10.4)**	**1.173 (1.04-1.259)**
		No GM	10 (77%)	8.3 (7.6-9.9)	8.7 (6.7-10.4)	1.099 (1.021-1.259)
		CGM	3 (23%)	7.9 (7.2-10.5)	6.5 (6.2-10.6)	1.259 (1.173-1.307)
	MDI	**Total**	**7**	**12.2 (6.7-13.2)**	**8.0 (6.1-9.4)**	**1.304 (1.211-1.367)**
		No GM	4 (57%)	12.7 (9.2-14.6)	7.9 (5.3-9.7)	1.257 (1.002-1.335)
		FGM	3 (43%)	10.1 (6.7-12.6)	8.7 (6.1-9.4)	1.336 (1.228-2.130)
MetS-	CSII	**Total**	**24**	**6.8 (6.2-8.1)**	**11.2 (9.6-11.7)**	**1.446 (1.213-1.644)**
		No GM	9 (38%)	7.8 (6.5-8.3)	10.8 (10.2-11.4)	1.414 (1.272-1.490)
		FGM	10 (42%)	6.9 (6.2-8.7)	11.5 (8.7-11.7)	1.596 (1.115-1.695)
		CGM	5 (20%)	6.4 (5.8-6.6)	11.7 (11.0-12.2)	1.419 (1.304-1.445)
	MDI	**Total**	**16**	**7.1 (6.6-11.6)**	**10.5 (7.7-11.0)**	**1.308 (1.225-1.455)**
		No GM	11 (69%)	11.2 (6.7-12.5)	10.4 (8.0-11.0)	1.322 (1.199-1.406)
		FGM	16 (31%)	6.6 (6.4-6.8)	10.8 (7.5-11.0)	1.294 (1.265-1.636)

Values are expressed as median and interquartile range (Me (Q_1-Q_3). Abbreviations: estimated glucose disposal resistance (eGDR), glucose monitoring (GM), glycated hemoglobin (HbA1c), continuous glucose monitoring (CGM), continuous subcutaneous insulin infusion (CSII), flash glucose monitoring (FGM), multiple daily injections (MDI), metabolic syndrome (MetS), total antioxidant status (TAS).

Table S2. TAS, HbA1c and eGDR values depending on insulin therapy and modern GM systems used.

Subgroup/ Category	CSII (n=37)	MDI (n=23)	No GM (n=34)	FGM (n=18)	CGM (n=8)
**TAS**					
**low**	18 (62%)	11 (38%)	19 (66%)	7 (24%)	3 (10%)
**medium**	17 (63%)	10 (37%)	14 (52%)	8 (30%)	5 (18%)
**high**	2 (50%)	2 (50%)	1 (25%)	3 (75%)	0 (0%)
**HbA1c**					
**HbA1c ≤7%**	15 (60%)	10 (40%)	8 (32%)	12 (48%)	5 (20%)
**HbA1c >7%**	22 (63%)	13 (37%)	26 (74%)	6 (17%)	3 (9%)
**eGDR**					
**≤8mg/min/kg**	7 (50%)	7 (50%)	9 (64%)	3 (21%)	2 (15%)
**>8mg/min/kg**	15 (35%)	30 (65%)	25 (54%)	15 (33%)	6 (13%)

Values are expressed as number and percentage of respondents (n(%)). Abbreviations: estimated glucose disposal resistance (eGDR), glucose monitoring (GM), glycated hemoglobin (HbA1c), continuous glucose monitoring (CGM), continuous subcutaneous insulin infusion (CSII), flash glucose monitoring (FGM), multiple daily injections (MDI), metabolic syndrome (MetS), total antioxidant status (TAS).

Table S3. Comparison of cardiovascular biomarkers depending on insulin therapy and modern GM systems used.

Subgroup/ Parameter	CSII (n=37)	MDI (n=23)	No GM (n=34)	FGM (n=18)	CGM (n=8)	p-value
**WC** (cm)	66 (62-73)	69 (66-74)	69 (62-74)	67 (64-74)	66 (62-71)	-
**WHR**	0.88 (0.81-0.91)	0.88 (0.84-0.91)	0.88 (0.83-0.92)	0.87 (0.81-0.9)	0.88 (0.84-0.9)	-
**WHtR**	0.42 (0.39-0.45)	0.42 (0.39-0.44)	0.42 (0.39-0.45)	0.42 (0.38-0.44)	0.42 (0.4-0.42)	-
**BMI** (kg/m^2)	20 (18-22)	21 (18-22)	20 (18-22)	20 (19-22)	20 (18-22)	-
**HbA1c** (%)	7.5 (6.5-8.8)	7.7 (6.6-12.5)	8.11 (7.12-11.24)	6.8 (6.31-8.65)	6.65 (6.1-7.54)	<0.001 F/CGM vs. no GM
**eGDR** (mg/kg/min)	9.4 (7.5-11)	10.5 (8.6-11.6)	10 (7.9-11)	10.7 (8.6-11.6)	10.8 (7.5-11.9)	-
**TC** (mg/dL)	151 (123-176)	144 (124-170)	142 (123-175)	156 (140-185)	152 (123-172)	-
**LDL-ch** (mg/dL)	83 (67-108)	90 (70-101)	83 (68-105)	85 (68-102)	92 (70-109)	-
**HDL-ch** (mg/dL)	56 (48-67)	48 (39-68)	50 (44-60)	63 (48-74)	59 (52-70)	<0.05 F/CGM vs. no GM
**TG** (mg/dL)	60 (49-81)	99 (56-119)	80 (58-117)	62 (52-99)	54 (46-78)	<0.05 MDI vs. CSII, F/CGM vs. no GM
**SBP** (mmHg)	116 (112-120)	113 (108-125)	114 (109-121)	117 (114-125)	114 (112-120)	-
**DBP** (mmHg)	71 (66-74)	72 (69-75)	72 (69-75)	72 (66-74)	69 (65-74)	-

Values are expressed as median and interquartile range (Me (Q_1-Q_3). Abbreviations: continuous glucose monitoring (CGM), continuous subcutaneous insulin infusion (CSII), diastolic blood pressure (DBP), estimated glucose disposal resistance (eGDR), flash glucose monitoring (FGM), high density lipoprotein cholesterol (HDL-ch), glucose monitoring (GM), glycated hemoglobin (HbA1c), low density lipoprotein cholesterol (LDL-ch), multiple daily injections (MDI), metabolic syndrome (MetS), systolic blood pressure (SBP), total cholesterol (TC), triglycerides (TG), waist circumference (WC), waist-hip ratio (WHR), waist-to-height ratio (WHtR).

Table S4. Comparison of body composition analysis parameters depending on insulin therapy and modern GM systems used.

Subgroup/ Parameter	CSII (n=37)	MDI (n=23)	No GM (n=34)	FGM (n=18)	CGM (n=8)
**Body weight** (kg)	53 (42-67)	56 (47-61)	56 (46-66)	53 (44-72)	51 (39-61)
**Body height** (cm)	164 (157-172)	168 (156-176)	167 (157-172)	163 (157-176)	158 (153-164)
**TBW** (L)	31 (27-35)	31 (27-39)	32 (28-38)	31 (27-42)	29 (24-31)
**SMM** (kg)	23 (19-26)	23 (20-30)	23 (20-29)	23 (20-32)	22 (17-23)
**Protein** (kg)	8 (7-9)	8 (7-11)	8 (7-10)	8 (7-11)	8 (6-8)
**Minerals** (kg)	3 (3-4)	3 (3-4)	3 (3-4)	3 (3-4)	3 (2-3)
**PBF** (%)	20 (14-29)	20 (12-26)	21 (15-29)	19 (12-26)	19 (14-26)
**VFA** (cm^2)	46 (30-67)	52 (35-76)	49 (35-76)	46 (26-59)	44 (32-62)

Values are expressed as median and interquartile range (Me (Q_1-Q_3). Statistically significant differences between the medians were not detected. Abbreviations: continuous glucose monitoring (CGM), continuous subcutaneous insulin infusion (CSII), flash glucose monitoring (FGM), glucose monitoring (GM), multiple daily injections (MDI), metabolic syndrome (MetS), percent body fat (PBF), skeletal muscle mass (SMM), total body water (TBW), visceral fat area (VFA).

Table S5. Consumption of the selected nutrients with diet depending on insulin therapy and modern GM systems used.

Nutrient	CSII (n=37)	MDI (n=23)	No GM (n=34)	FGM (n=18)	CGM (n=8)
	Me ± IQR				
			**Main nutrients**		
**Energy (kcal)**^{A*}	1803 (1685-1944)	1793 (1631-1904)	1804 (1631-1927)	1831 (1726-1934)	1740 (1533-1809)
**Protein (%TDEE)**	18 (16-21)	19 (18-21)	19 (17-21)	16 (15-18)	21 (20-22)
**Carbohydrate (%TDEE)**	57 (51-61)	54 (51-56)	54 (50-59)	58 (53-61)	55 (52-57)
**Fat (%TDEE)**	22 (20-28)	25 (22-29)	25 (21-30)	22 (21-29)	23 (21-25)
			**Fatty acids**		
**SFA (g)**	17 (15-20)	16 (14-18)	16 (15-20)	17 (15-19)	17 (15-18)
**Palmitic FA (g)**	10 (9-11)	11 (9-12)	10 (9-11)	10 (9-12)	11 (10-12)
**MUFA (g)** ^{B*}	13 (11-18)	16 (13-22)	14 (12-20)	16 (11-23)	11 (8-14)
**Oleic FA (g)**	13 (10-14)	14 (12-17)	13 (11-17)	14 (11-15)	13 (9-13)
**PUFA (g)**	5 (4-8)	6 (6-8)	6 (5-8)	6 (4-9)	5 (3-6)
**LC-PUFA (g)**	0.052 (0.039-0.090)	0.069 (0.052-0.159)	0.066 (0.031-0.104)	0.054 (0.039-0.09)	0.069 (0.045-0.217)
**ω-3 (g)** ^{B*}	0.688 (0.554-0.975)	0.789 (0.566-1.336)	0.785 (0.573-1.173)	0.693 (0.535-1.399)	0.688 (0.565-1.162)
**ALA (g)**	0.554 (0.469-0.788)	0.607 (0.505-1.03)	0.626 (0.484-0.851)	0.544 (0.442-1.03)	0.55 (0.339-0.635)
**EPA (g)**	0.012 (0.004-0.016)	0.015 (0.009-0.04)	0.012 (0.004-0.026)	0.012 (0.01-0.015)	0.012 (0.011-0.041)
**DHA (g)**	0.029 (0.021-0.061)	0.038 (0.025-0.101)	0.037 (0.018-0.067)	0.034 (0.021-0.067)	0.039 (0.026-0.144)
**ω-6 (g)**	5 (4-6)	6 (5-7)	5 (4-6)	6 (4-7)	4 (3-5)
**LA (g)** ^{B*}	5 (4-6)	5 (4-7)	5 (4-6)	6 (4-7)	4 (3-5)
**AA (g)**	0.042 (0.031-0.08)	0.054 (0.034-0.153)	0.045 (0.03-0.141)	0.051 (0.034-0.083)	0.044 (0.033-0.157)
			**Carbohydrates**		
**Glucose (g)**	5 (3-9)	5 (3-7)	4 (3-6)	5 (5-8)	8 (2-11)
**Fructose (g)**	8 (4-12)	7 (5-11)	6 (4-12)	8 (6-13)	11 (3-13)
**Saccharose (g)**	34 (15-51)	33 (14-47)	34 (13-54)	33 (17-47)	34 (20-36)
**Dietary fiber (g)** ^{A,B**}	18 (13-21)	17 (14-22)	17 (13-20)	21 (18-23)	13 (12-17)

Values are expressed as median and interquartile range (Me (Q_1-Q_3). Statistically significant differences (*$p<0.05$, **$p<0.001$) between the medians were marked as "A" (FGM vs. no GM) or "B" (CGM vs. no GM). Abbreviations: arachidonic acid (AA), alpha-linolenic acid (ALA), continuous glucose monitoring (CGM), continuous subcutaneous insulin infusion (CSII), docosahexaenoic acid (DHA), eicosapentaenoic acid (EPA), fatty acids (FA), flash glucose monitoring (FGM), glucose monitoring (GM), linoleic acid (LA), multiple daily injections (MDI), long-chain polyunsaturated fatty acids (LC-PUFA), metabolic syndrome (MetS), monounsaturated fatty acids (MUFA), polyunsaturated fatty acids (PUFA), saturated fatty acids (SFA), total daily energy expenditure (TDEE).
